# Pharmacological Effects of Methotrexate and Infliximab in a Rats Model of Diet-Induced Dyslipidemia and Beta-3 Overexpression on Endothelial Cells

**DOI:** 10.3390/jcm10143143

**Published:** 2021-07-16

**Authors:** Denisa-Mădălina Zălar, Cristina Pop, Elena Buzdugan, Bela Kiss, Maria-Georgia Ştefan, Steliana Ghibu, Valentin-Adrian Bâlteanu, Doiniţa Crişan, Alexandra Buruiană-Simic, Adriana Grozav, Cristina Ionela Mogoșan

**Affiliations:** 1 Department of Pharmacology, Physiology and Pathophysiology, Faculty of Pharmacy, “Iuliu Hațieganu” University of Medicine and Pharmacy, 400349 Cluj-Napoca, Romania; denisazalar@yahoo.com (D.-M.Z.); steliana.ghibu@umfcluj.ro (S.G.); cmogosan@umfcluj.ro (C.I.M.); 2 Department of Cardiology, 5th Medical Clinic, Faculty of Medicine, “Iuliu Hațieganu” University of Medicine and Pharmacy, 400012 Cluj-Napoca, Romania; buzelena@yahoo.com; 3 Department of Toxicology, Faculty of Pharmacy, “Iuliu Hațieganu” University of Medicine and Pharmacy, 400012 Cluj-Napoca, Romania; kbela@umfcluj.ro (B.K.); m.georgia.stefan@gmail.com (M.-G.Ş.); 4 Faculty of Animal Science and Biotechnologies, Institute of Life Sciences, University of Agricultural Sciences and Veterinary Medicine Cluj-Napoca, 400372 Cluj-Napoca, Romania; lzga.usamvcj@yahoo.com; 5 Department of Pathology, Faculty of Medicine, “Iuliu Hațieganu” University of Medicine and Pharmacy, 400012 Cluj-Napoca, Romania; dcrisan@umfcluj.ro (D.C.); buruiana.alexandra@yahoo.com (A.B.-S.); 6 Department of Organic Chemistry, Faculty of Pharmacy, “Iuliu Hațieganu” University of Medicine and Pharmacy, 400012 Cluj-Napoca, Romania; adriana.ignat@umfcluj.ro

**Keywords:** high-lipid diet, transgenic rats, beta-3 receptor overexpression, inflammation, oxidative stress, aortic thickening, methotrexate, infliximab

## Abstract

Background: Hyperlipidemia and inflammation are critical components in the pathophysiology of endothelial disorder, which can lead to vascular complications. Our study aimed to evaluate the effects of immunomodulatory therapy (methotrexate and infliximab) in a diet-induced hyperlipidemia rat model. Methods: Sprague-Dawley (wild type (WT), male, *n* = 32) rats were divided into four groups: one group fed with standard diet (SD), one group fed with high lipid diet (HLD), and two groups that received HLD and drug treatment (methotrexate (Mtx) or infliximab (Ifx)). In order to evaluate if modifications to the endothelial cells may influence the risk of vascular complications following hyperlipidemia or treatment reactivity, each group was doubled by a rats group that overexpressed beta-3 receptors on the endothelial cells (transgenic (TG-beta 3), male, *n* = 32). Serum lipid profile, liver enzymes, oxidative stress, and inflammation markers were determined. Histopathologic analysis of the liver and aorta was performed. Results: After 9 weeks of HLD, rats exhibited significant pathologic serum lipid profiles, elevated oxidative stress, and pro-inflammatory markers. Additionally, the aortic histopathological analysis revealed aorta media-intima thickening (*p* < 0.05) in the transgenic group. Methotrexate and infliximab significantly decreased inflammation and oxidative stress parameters, but presented opposing effects on lipid profiles (methotrexate decreased, whereas infliximab increased the atherosclerosis index). Drug treatment decreased the aorta media-intima thickness (*p* < 0.05) only in transgenic rats. Conclusions: HLD was associated with hyperlipidemia, inflammation and oxidative stress. The overexpression of beta-3 receptors on endothelial cells increased aortic thickening in response to the HLD. Methotrexate and infliximab reduced oxidative stress and inflammation in all groups, but led to favorable histopathologic vascular results only in the transgenic groups.

## 1. Introduction

Atherosclerotic cardiovascular disease is considered one of the leading causes of mortality worldwide. Based on *National Health and Nutrition Examination Survey* (NHANES) (2013 to 2016), the prevalence of cardiovascular disease (CVD) including coronary artery disease (CAD), heart failure (HF), stroke, and hypertension in adults ≥20 years old was 48.0% (121.5 million out of 2016). Prevalence of CVD increases with age in both genders [1].

Currently, atherosclerosis is characterized by the activation of inflammatory pathways, with many studies highlighting the involvement of inflammation in all stages of atherogenesis [2,3]. For a long time, atherosclerosis was regarded as a disease defined by the lipoproteins deposition in the arterial wall [4,5,6], however, in recent decades the increasing research has changed the viewpoint regarding the atherosclerotic process revealing that dyslipidemia and inflammation are critical factors in the onset, progression, and rupture of atherosclerotic plaques [7,8,9]. Pro-inflammatory cytokines represent a significant part of atherosclerosis as a chronic inflammatory disease. In recent studies, high C-reactive protein levels (CRP) have been regarded as an indicator of future cardiovascular events, becoming measurable in the first phases of atherogenesis. In locally damaged tissue, activated macrophages release many pro-inflammatory cytokines interleukine-1β (IL-1β), interleukine-6 (IL-6), and tumor necrosis factor-α (TNF-α), promoting high CRP levels to be synthesized by the liver [10,11]. IL-1β, IL-6, TNF-α, and CRP were previously evaluated for their potential to be therapeutic targets for anti-inflammatory treatment in atherosclerosis, thus reducing the residual risk of atherosclerotic cardiovascular disease [12].

Methotrexate and infliximab were recently studied for immuno-modulatory properties, targeting inflammatory pathways activated in different cardiovascular diseases [13].

Methotrexate is a folic acid antagonist that belongs to the class of cytotoxic drugs known as antimetabolites and works by competitively inhibiting the enzyme dihydrofolate reductase and thus inhibiting DNA synthesis. Recent studies showed that in patients with rheumatoid arthritis (RA), methotrexate administration diminished CVD risk by reducing several pro-inflammatory cytokines (IL-6, TNF-α) and CRP [14,15], purine biosynthesis, and may also stimulate the release of adenosine, all of which may lead to its anti-inflammatory properties [16,17].

Infliximab is a monoclonal antibody that binds with high affinity to soluble and transmembrane forms of TNFα. In vitro, infliximab has shown to inhibit the functional activity of TNFα. In vivo, infliximab forms stable complexes with human TNFα, a process that leads to loss of TNFα bioactivity. Bernatsky et al. [18] recently reported a decline of CVD in RA patients treated with TNF-α antagonist, supporting the possibility that TNF-α inhibitors could be useful in reducing the CVD risk [18,19,20].

Several animal models of atherosclerosis have been developed [21], but the most reproducible are the ones using high lipid diets [21]. Although it is difficult to develop typical atherosclerosis in rats, studies show that a diet rich in lard and cholesterol administered to rats for more than 4 weeks can induce dyslipidemia, inflammation, and gradually lead to vascular modifications resulting in atherosclerosis-like lesions [22]. Atherosclerosis is associated with endothelial dysfunction and stiffening of the arterial wall. Thus, the overexpression of beta-3 receptors at the endothelial level could lead to increased endothelial dysfunction [22], an enhanced vascular reactivity to hyperlipidemia, and vascular modifications indicative of atherosclerosis development.

Thus, we aimed to investigate the effects of methotrexate and infliximab in an animal model of diet-induced dyslipidemia and overexpression of beta-3 receptor on endothelial cells.

## 2. Materials and Method

### 2.1. Animals, Substances and Experimental Design

Thirty-two healthy adult male Sprague-Dawley (wild type, WT) rats, weighing 300 ± 20 g were purchased from “Cantacuzino” National Medico-Military Institute for Research and Development, Bucharest, Romania, and 32 healthy adult male transgenic Sprague-Dawley rats that exhibit overexpression of endothelial cell beta-3 receptors (TG-beta 3), weighing 280 ± 20 g, were provided by Therassay laboratories, France.

All techniques and procedures were performed according to the National Institutes of Health Guide for the Care and Use of Laboratory Animals [23] and were approved by the Ethics Committee of University of Medicine and Pharmacy “Iuliu Hatieganu” Cluj-Napoca (Permit number: 162/10.04.2019).

The rats were housed in an environmentally controlled room at 23 ± 1 °C and 55 ± 5% relative humidity, with a 12-h light/dark cycle. Food and water were provided ad libitum. All efforts were made to minimize the suffering of rats.

Drugs used in the experiment were commercially available: methotrexate (Sigma-Aldrich, Co., St. Louis, MO, USA, batch number: BCBV8405) and infliximab (drug approval number: EU/1/13/854/001; batch number: 232192).

The rats’ standard diet was purchased from “Cantacuzino” National Medico-Military Institute for Research and Development, Bucharest, Romania. High-lipid diet consisted of 75% basal diet, 9% lard, 5.5% yolk powder, 7.5% sugar, 2.5% cholesterol, 0.3% sodium cholate, and 0.2% propylthiouracil [24].

After 1 week of acclimatization, rats were weighed and randomly divided into eight groups of eight rats per group as follows: 

(1) wild-type with standard diet—WT + SD group; (2) transgenic with standard diet—TG-beta 3 + SD group; (3) wild type with high-lipid diet—WT + HDL; (4) transgenic with high-lipid diet—TG-beta 3 + HDL; (5) wild-type with high-lipid diet and methotrexate treatment—WT + HLD + Mtx (0.1 mg/kg, i.p., daily administration); (6) transgenic with high-lipid diet and methotrexate treatment TG-beta3 + HLD + Mtx (0.1 mg/kg, i.p., daily administration); (7) wild-type with high-lipid diet and infliximab treatment—WT + HLD + Ifx (1.5 mg/kg, subcutaneous (s.c.) weekly administration); (8) transgenic with high-lipid diet and infliximab treatment—TG-beta 3 + HLD + Ifx (1.5 mg/kg, subcutaneous (s.c.) weekly administration).

All rats were fed the specific diet (SD or HLD) for 9 weeks. For the treatment groups, drugs were included in the protocol after 4 weeks of the diet. The treatment period was 5 weeks.

At the end of the study, blood was collected through retro-orbital sinus puncture under anesthesia with 30 mg/kg ketamine and 0.5 mg/kg xylazine, i.m. Blood samples were centrifuged at 3500 RPM for 15 min, and plasma was separated and stored at −80 °C until analysis. Afterwards, animals were sacrificed by cervical dislocation. Liver, aorta, and myocardium tissue samples were harvested and fixed in 10% formalin at 4°C until assayed.

### 2.2. Biochemical Analysis

Plasma levels of cholesterol (TC), triglyceride (TG), low-density lipoprotein cholesterol (LDL-C), and high-density lipoprotein cholesterol (HDL-C), were determined using a spectrophotometer analyzer (Cobas Integra 400 Plus (Roche Diagnostics, Basel, Switzerland). Liver function parameters such as aspartate aminotransferase (AST) and alanine aminotransferase (ALT) were also measured. The arteriosclerosis index (AI) was calculated as follows: [24]

(1) $$AI=\frac{TC-HDL\;Cholesterol}{HDL\;Cholesterol}$$

### 2.3. Determination of Inflammation Markers 

The concentrations of hs-CRP, IL-1β, IL-6, and TNF-α in plasma were measured using specific ELISA kits according to the manufacturer’s instructions (Elabscience USA and EIAab China).

### 2.4. Determination of Oxidative Stress Markers

Plasma samples required for reduced glutathione (GSH), oxidized glutathione (GSSG), and malondialdehyde (MDA) analysis were obtained by centrifugation of EDTA-treated whole blood.

One-hundred-and-fifty microliters of plasma was deproteinized with 10% metaphosphoric acid (m/v). After dilution with 0.1% EDTANa_2_ in phosphate buffer (pH = 8), a fraction of the supernatant was incubated with ortho-phthalaldehyde (OPA), followed by chromatographic assay of derivatized GSH. In the case of GSSG, the deproteinization was followed by incubation with N-ethylmaleimide, dilution with 0.1 M NaOH, derivatization with OPA, and chromatographic assay.

The chromatographic system consisted of a Waters Acquity UPLC system coupled with Waters Acquity fluorescence detector (Waters, Milford, MA, USA) (λ_exc_ = 350 nm, λ_em_ = 420 nm). The separation was achieved in both cases using an HSS T3 Acquity UPLC column (1.8 µm, 2.1 × 100 mm) and a mixture of Na_2_HPO_4_ 25 mM and MeOH as the mobile phase.

For total MDA analysis, alkaline hydrolysis was achieved by incubation of plasma with 6 M NaOH at 60 °C in a water bath, followed by the addition of the internal standard (diacetone alcohol), deproteinization with 35% perchloric acid, and derivatization with 2,4-dinitrophenylhydrazine. The obtained derivative was extracted in hexane, followed by the evaporation to dryness of the organic phase. The residue was dissolved in the mobile phase and injected in the UPLC-PDA system (λ = 307 nm). The separation was performed on a BEH C18 column (50 mm × 2.1 mm i.d., 1.7 mm), using gradient elution (1% formic acid/acetonitrile). Empower two software (Waters, Milford, MA, USA) was used for data acquisition and processing in the case of all chromatographic assays.

### 2.5. Organ Index of the Liver, Kidney and Myocardium

The organ index of liver, kidney, and myocardium were calculated applying the following formula: organ index (%) = (organ weight/body weight) × 100% [25].

### 2.6. Histopathological Analysis of Liver, Heart and Aorta

Paraffin sections of aortic arch, liver, and heart (4–5 µm) were sliced using Microtome Microtec CUT 4050 (Walldorf, Germany) and stained with hematoxylin-eosin (H&E); frozen sections of the aortic arch and liver (10 µm) were sliced using the Criotom Leica CM 1860 UV and stained with Oil Red. The images were captured on a Leica DM750 microscope with Leica ICC 50 HD Camera, using the LAS V4.12. image analyzer. Further, the sections (aortic root, liver) were analyzed and the percentage of foam cells in the liver in every random field was quantified. Aorta, intima, and media thickness in every random field were also quantified. Regarding the liver’s histopathological analysis, we classified the liver disease based on liver architecture, steatosis liver score, lobular inflammation score, ballooning score, and grading of steatohepatitis.

### 2.7. Statistical Analysis

The statistical analysis was performed using IBM SPSS 26.0 (Statistical Package for the Social Science, SPSS Inc., Chicago, IL, USA) software and GraphPad Prism 5.0 (GraphPad Software, San Diego, CA, USA). All experimental data were represented as mean ± SD. Data were compared using Student’s *t*-tests for quantitative variables, Mann–Whitney tests for ordinal/semiquantitative variables, Fisher tests for qualitative variables, and One-way ANOVA for multi-group comparisons. *p* < 0.05 was considered significant.

## 3. Results

### 3.1. Effects on Plasma Lipid Levels

**Figure 1:**
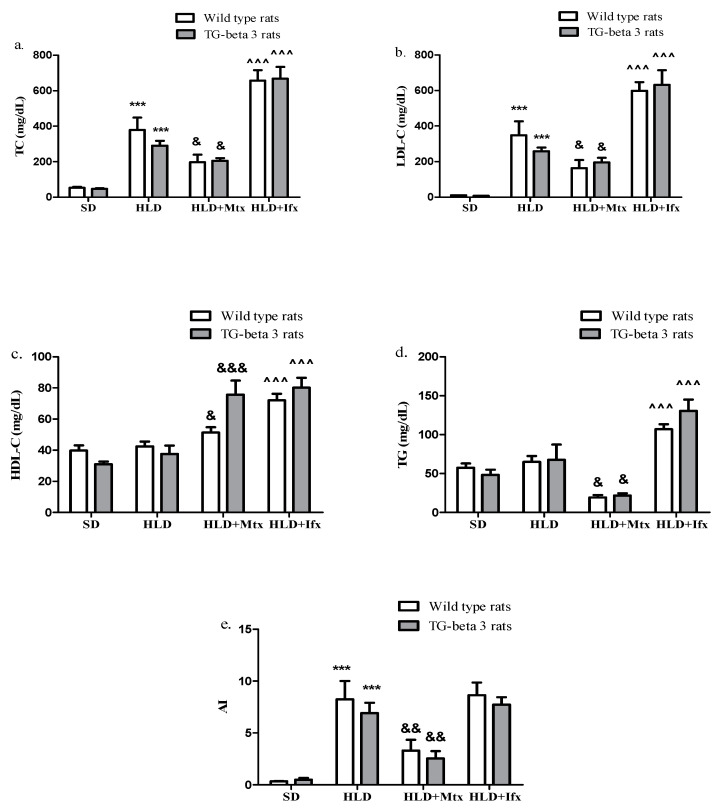
Methotrexate and infliximab effects on plasma lipid levels. (**a**) Total cholesterol (TC), (**b**) low-density lipoprotein cholesterol (LDL-C), (**c**) high-density lipoprotein cholesterol (HDL-C), (**d**) triglycerides (TG). (**e**) atherosclerosis index (AI). SD—standard diet; HLD—high lipid diet; Mtx-methotrexate; Ifx-infliximab; HLD + Mtx—high lipid diet associated with methotrexate; HLD + Ifx—high lipid diet associated with infliximab. *** *p* < 0.001 HLD versus SD group; ^&^ *p* < 0.05; ^&&^ *p* < 0.01; ^&&&^ *p* < 0.001 HLD + Mtx versus HLD group; ^^^^^ *p* < 0001 HLD + Ifx versus HLD group.

After 9 weeks of HLD, TC, LDL-C, and AI (>4) were significantly (*p* < 0.001) higher in HLD groups compared to SD groups (Figure 1a,b,e). HDL-C and triglycerides levels across the SD and HLD groups were comparable (Figure 1c,d).

Methotrexate administration showed a significant (*p* < 0.05) decrease in TC, LDL-C, TG, and AI levels (*p* < 0.01), while HDL-C significantly increased (Figure 1).

The treatment with infliximab significantly (*p* < 0.001) increased the lipid profile (TC, LDL-C, HDL-C, and TG), but did not influence the AI (Figure 1). There were no significant differences between the wild type and transgenic-beta 3 rats regarding the lipid profile and AI (*p* > 0.05) (Figure 1).

### 3.2. Inflammation Markers

**Figure 2:**
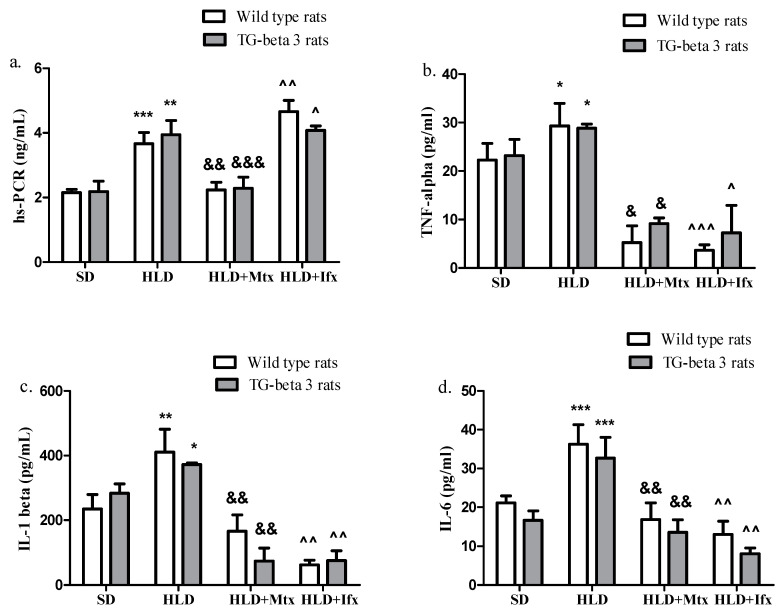
Methotrexate and infliximab effects on inflammation markers. (**a**) High sensitivity reactive protein C (hs-PCR), (**b**) tumor necrosis factor-alpha (TNF-alpha), (**c**) interleukine-1 beta (IL-1 beta), (**d**) interleukine-6 (IL-6). SD—standard diet; HLD—high lipid diet; Mtx-methotrexate; Ifx-infliximab; HLD + Mtx—high lipid diet associated with methotrexate; HLD + Ifx—high lipid diet associated with infliximab. * *p* < 0.05; ** *p* < 0.01; *** *p* < 0.001 HLD versus SD group; ^&^ *p* < 0.05; ^&&^ *p* < 0.01; ^&&&^ *p* < 0.001 HLD + Mtx versus HLD group; ^^^ *p* < 0.05; ^^^^ *p* < 0.01; ^^^^^ *p* < 0.001 HLD + Ifx versus HLD group.

As shown in Figure 2a, inflammation markers considerably increased after the HLD, while methotrexate administration led to a significant decrease in these parameters (hs-PCR, TNF-α, IL-1β and IL-6). Under treatment with infliximab, the hs-PCR levels showed a slight increase, while TNF-α, IL-1β, and IL-6 considerably decreased. There were no significant differences between the wild type and transgenic-beta 3 rats regarding the inflammation markers (*p* > 0.05).

### 3.3. Oxidative Stress Markers

**Figure 3:**
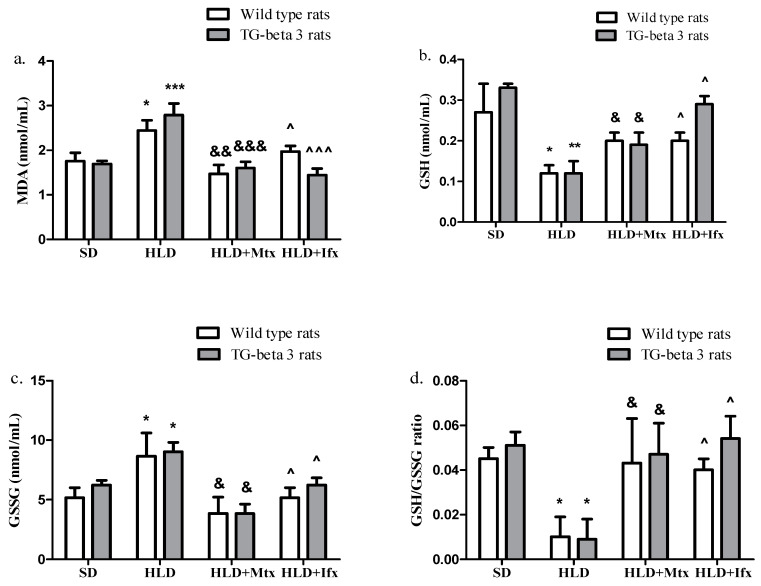
Methotrexate and infliximab effects on oxidative stress markers. (**a**) Malondialdehyde (MDA), (**b**) reduced glutathione (GSH); (**c**) oxidized glutathione (GSSG); (**d**) GSH/GSSG ratio. SD—standard diet; HLD—high lipid diet; Mtx-methotrexate; Ifx-infliximab; HLD + Mtx—high lipid diet associated with methotrexate; HLD + Ifx—high lipid diet associated with infliximab. * *p* < 0.05; ** *p* < 0.01; *** *p* < 0.001 HLD versus SD group; ^&^ *p* < 0.05; ^&&^ *p* < 0.01; ^&&&^ *p* < 0.001 HLD + Mtx versus HLD group; ^^^ *p* < 0.05; ^^^^^ *p* < 0.001 HLD + Ifx versus HLD group.

The oxidative stress biomarkers, such as MDA and GSSG levels, were increased in HLD groups than SD groups (Figure 3a,c). GSH and GSH/GSSG ratio were lower in HLD groups than SD groups (Figure 3b,d). 

In the methotrexate and infliximab-treated groups, MDA and GSSG levels significantly decreased compared to HLD groups (Figure 3a,c), while GSH and GSH/GSSG ratio markedly (*p* < 0.05) increased (Figure 3c,d). There were no significant differences between the wild type and transgenic-beta 3 rats regarding the oxidative stress markers (*p* > 0.05).

### 3.4. Effects on Liver Function Enzymes

**Figure 4:**
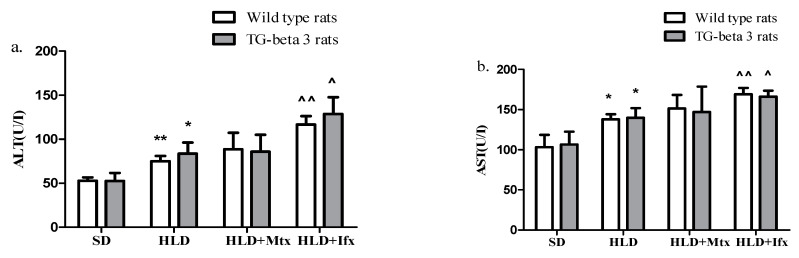
Methotrexate and infliximab effects on liver function. (**a**) Alanine aminotransferase (ALT), (**b**) aspartate aminotransferase (AST). SD—standard diet; HLD—high lipid diet; Mtx-methotrexate; Ifx-infliximab; HLD + Mtx—high lipid diet associated with methotrexate; HLD + Ifx—high lipid diet associated with infliximab. * *p* < 0.05; ** *p* < 0.01 HLD versus SD group; ^^^ *p* < 0.05; ^^^^ *p* < 0.01 HLD + Ifx versus HLD group.

Transaminases (ALT and AST) presented a significant increase in HLD groups compared to SD groups in both wild type and transgenic-beta 3 rats (Figure 4a,b). Methotrexate treatment did not influence transaminase levels in any group. At the same time, infliximab led to significantly increased ALT and AST levels in both wild type and transgenic-beta 3 rats (Figure 4a,b).

### 3.5. Histopathological Analysis

**Figure 5:**
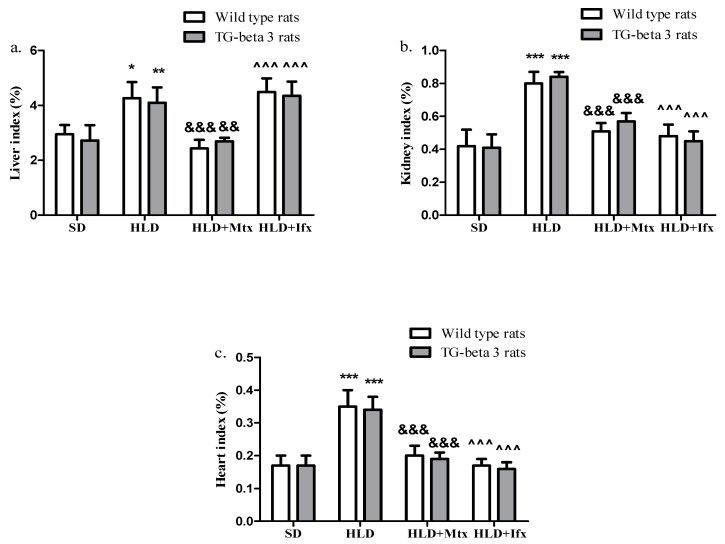
Organ index of liver, kidney and heart. (**a**) Liver index; (**b**) kidney index; (**c**) heart index. SD—standard diet; HLD —high lipid diet; Mtx-methotrexate; Ifx-infliximab; HLD + Mtx—high lipid diet associated with methotrexate; HLD + Ifx—high lipid diet associated with infliximab. * *p* < 0.05; ** *p* < 0.01; *** *p* < 0.001 HLD versus SD group; ^&&^ *p* < 0.01; ^&&&^ *p* < 0.001 HLD + Mtx versus SD group; ^^^^^ *p* < 0.001 HLD + Ifx versus SD group.

The histopathological analysis revealed that the liver index was considerably higher in HLD groups than SD groups (Figure 5a). Methotrexate considerably decreased the liver index, while infliximab administration led to an increased liver index in both types of animals (Figure 5a). Kidney and heart index increased (*p* < 0.001) in HLD groups compared to SD groups (Figure 5b,c). At the same time, the treatment with methotrexate and infliximab showed a significant (*p* < 0.001) decrease in kidney and heart index values across groups (Figure 5b,c). There were no significant differences regarding the liver, kidney, and heart index between the wild type and transgenic-beta 3 rats (*p* > 0.05). 

**Figure 6:**
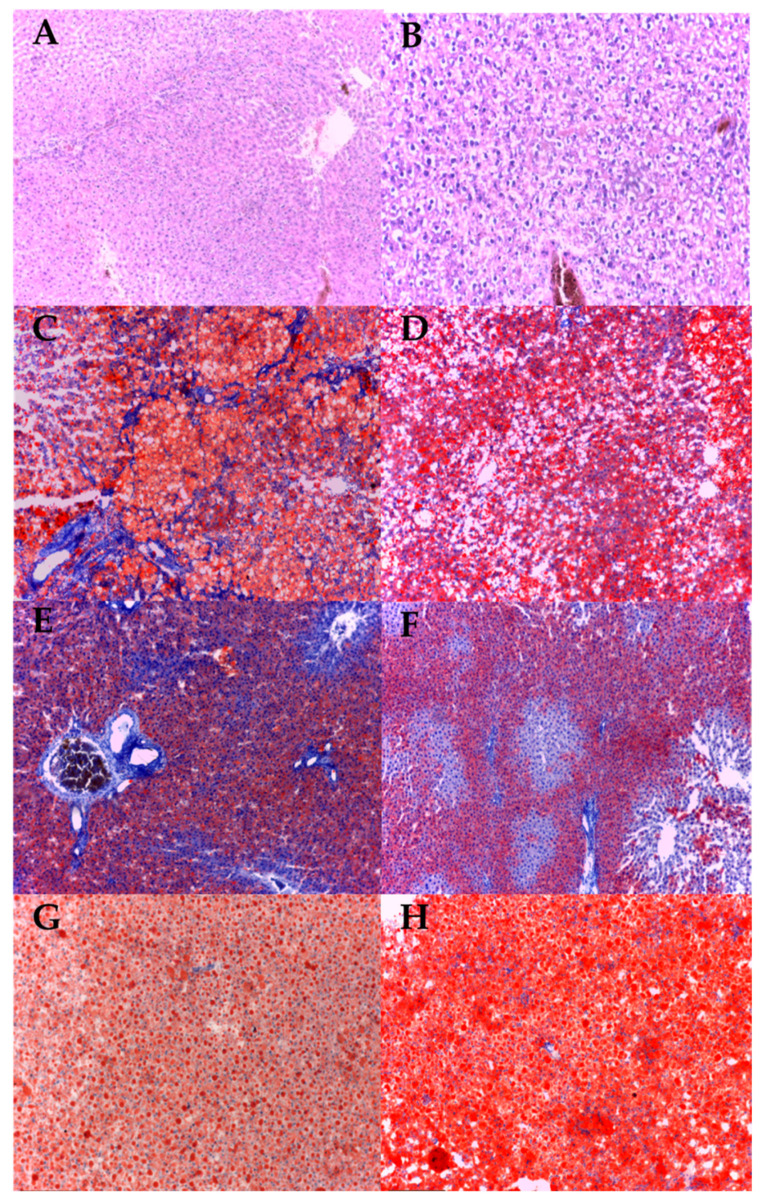
Photomicrographs of liver sections stained with Red Oil staining (200X). The red coloration indicates lipid droplets. (**A**) TG-beta 3 + SD, (**B**) WT + SD, (**C**) TG-beta 3 + HLD, (**D**) WT + HLD, (**E**) TG-beta 3 + HLD + Mtx, (**F**) WT + HLD + Mtx, (**G**) TG-beta 3 + HLD + Ifx, (**H**) WT + HLD + Ifx. WT-wild type; TG-beta 3- transgenic beta 3; SD—standard diet; HLD—high lipid diet; HLD + Mtx—high lipid diet associated with methotrexate; HLD + Ifx—high lipid diet associated with infliximab.

**Figure 7:**
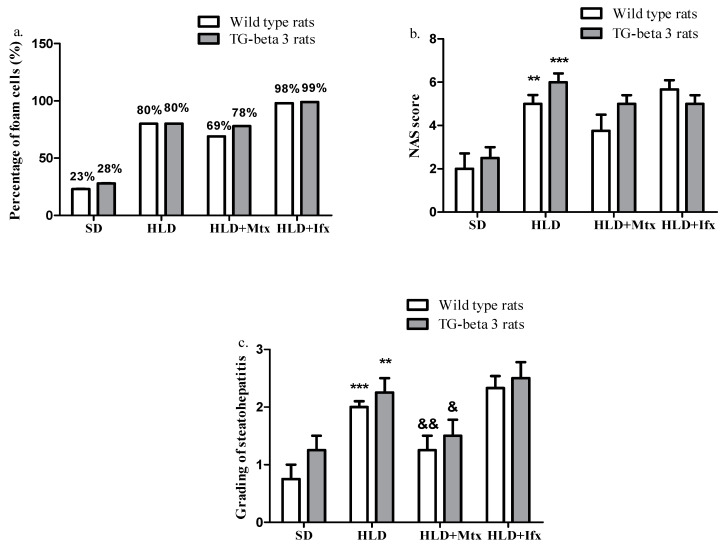
Histopathological analysis of liver. (**a**) Percentage of foam cells in the liver, (**b**) non-alcoholic fatty liver disease activity score (NAS), (**c**) grading of steatohepatitis. 1—mild, 2—moderate, 3—severe. SD—standard diet; HLD—high lipid diet; Mtx-methotrexate; Ifx-infliximab; HLD + Mtx—high lipid diet associated with methotrexate; HLD + Ifx—high lipid diet associated with infliximab. ** *p* < 0.01; *** *p* < 0.001 HLD versus SD group; ^&^ *p* < 0.05; ^&&^ *p* < 0.01 HLD + Mtx versus SD group.

Histopathological examination revealed that the high lipid diet led to a significant accumulation of lipid droplets in the intercellular and intracellular compartment of liver cells compared to those that received a standard diet. Methotrexate-treated groups showed a slight decrease in hepatic lipid accumulation compared to the HLD groups, while under infliximab treatment, hepatic lipid accumulation increased compared with HLD groups (Figure 6 and Figure 7a). There were no significant differences between the wild type and transgenic-beta 3 rats regarding the percentage of foam cells in the liver (*p* > 0.05) (Figure 7a).

Based on the sum of scores for each lesion (steatosis, lobular inflammation and hepatocellular ballooning), we determined the non-alcoholic fatty liver disease (NAFLD) activity score (NAS). The groups that received HLD presented a higher (*p* < 0.05) NAS score than those that received the SD. There is a decreasing trend for NAS score in the Mtx-treated group without statistical significance, while infliximab did not influence the NAS score (Figure 7b). There were no significant differences between the wild type and transgenic-beta 3 rats regarding the NAS score (*p* > 0.05).

In the histopathological liver sections, we found that steatohepatitis grading evolved in the groups fed with the high-lipid diet compared to the standard diet from mild to moderate (*p* < 0.05). Under treatment with methotrexate, the degree of steatohepatitis decreased significantly, while infliximab did not influence the steatohepatitis grading (Figure 7c). There were no significant differences between the wild type and transgenic-beta 3 rats regarding the steatohepatitis grading (*p* > 0.05).

**Figure 8:**
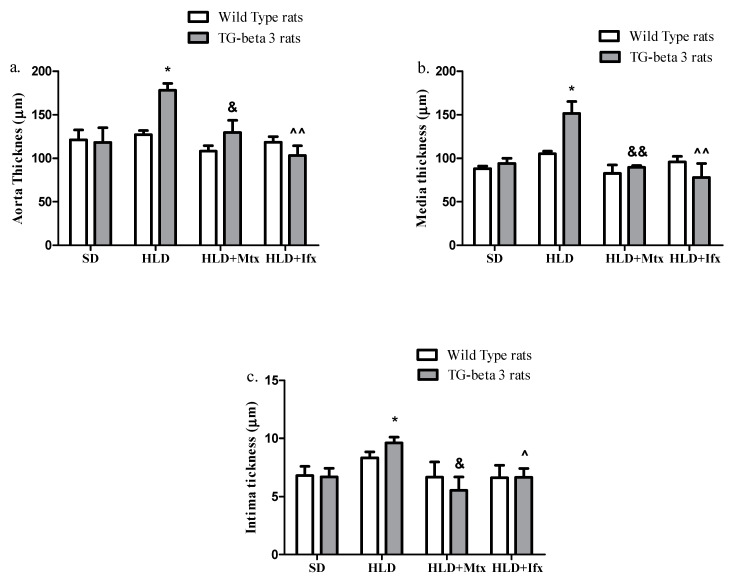
Methotrexate and infliximab effects on aortic histopathological sections. (**a**) Aorta thickness; (**b**) media thickness; (**c**) intima thickness. SD—standard diet; HLD—high lipid diet; Mtx-methotrexate; Ifx-infliximab; HLD + Mtx—high lipid diet associated with methotrexate; HLD + Ifx—high lipid diet associated with infliximab. * *p* < 0.05; HLD versus SD group; ^&^ *p* < 0.05; ^&&^ *p* < 0.01; HLD + Mtx versus SD group; ^^^ *p* < 0.05; ^^^^ *p* < 0.01; HLD + Ifx versus SD group.

In the transgenic-beta 3 group, we observed high-lipid diet-induced media and intima thickening (*p* < 0.05) in the aortic histopathological sections (Figure 8); deposition of foam cells in the sections stained with hematoxylin-eosin and oil red; a disordered arrangement of the nucleus; and the presence of smooth muscle cells and elastic fibers. In contrast, in the wild type group, no differences were observed. Treatment with methotrexate and infliximab produced a decrease in the media (*p* < 0.01) and intima (*p* < 0.05) thickness in transgenic rats, without modifications in wild type rats (Figure 8).

## 4. Discussions

Dyslipidemia is one of the leading causes of premature coronary atherosclerosis, leading to ischemic heart disease (IHD). Hypercholesterolemia with elevated LDL-C and low HDL-C are linked to increased incidence of CAD and cerebrovascular morbidity and mortality [26,27]. We aimed to evaluate the potential effects of methotrexate and infliximab on the lipid profile, inflammatory and oxidative processes, and also on the aortic wall in an animal model of dyslipidemia and beta-3 receptor overexpression on the endothelial cells. 

In the present study, we induced dyslipidemia by feeding Sprague-Dawley (wild type, WT) rats and Sprague-Dawley rats that exhibit overexpression of endothelial cell beta-3 receptors (TG-beta 3) with a high-lipid-diet for 9 weeks. Animal models that mimic atherosclerosis development by dyslipidemia have been studied to elucidate the pathogenesis and possible therapeutic options for this condition. Among animal models of atherosclerosis, the best-characterized are small rodents, rabbits, and pigs [21]. Most rodent models use high-lipid diets, mechanical injury, chemical injury, genetic manipulations, or associations of synergistic comorbidities (diabetes, hypertension etc.) [21,28]. In our particular model, we used a diet rich in lipids comprising a combination of lard, bile acid salts to improve fatty acids absorption and cholesterol with propylthiouracil, an anti-thyroid drug, reducing lipid-oxidation and enhancing the cholesterol level [24]. Although dyslipidemia developed after about 4 weeks, the total duration of the study (i.e., exposure to high-lipid diet) was 9 weeks. Commonly, rats are resistant to atherosclerosis because normally HDL-C is the dominant lipoprotein that leads to an increased cholesterol hepatic transport [21]. However, with specific interventions, rat-dyslipidemia and atherosclerotic vascular modifications can be achieved [28]. Another advantage of rats is their very well characterized genome, which allows for relatively easy genetic manipulation [28]. Genetic models of atherosclerosis include Apo-E knockout mice and rats, impaired fibrillin gene, or LDL-receptor deficiency [21]. Although costs are relatively high, their advantage is the direct etiologic link to atherosclerosis. Also, other genetic interventions with the purpose of inducing co-morbidities in order to better mimic the development of complex diseases can be made [21]. The overexpression of beta-3 adrenoreceptors in endothelial cells can increase the risk of endothelial dysfunction and together with dyslipidemia may accelerate vascular modifications and lesions [22]. Chemically-induced atherosclerosis can be obtained with Triton WR-1339, bisphenol-A, and poloxamer 407 [29,30]. Although quite effective, these substances do not mimic the long-term effects of dyslipidemia on the arterial wall, as it develops in humans and does not allow long-term treatment efficacy evaluation.

In our study, the HLD induced hyperlipidemia, which is highlighted by higher serum levels of TC, LDL-C, and initiated an inflammatory process shown by higher serum levels of inflammation markers (hs-PCR, TNF-α, IL-1β, IL-6) in the HLD group compared to the group fed with SD. The HLD triggers endothelial dysfunction characterized by an imbalance between vasodilation mediated by nitric oxide (NO) and prostacyclin-2 (PGI2) and vasoconstriction mediated by endothelin-1 (ET-1), causing abnormal responses that initiate atherosclerosis [31,32]; these implicate raised endothelial permeability, platelet aggregation, leukocyte adhesion, and cytokines release [31,32]. The LDL accumulated in the subendothelial space will be oxidized by reactive oxygen species (ROS) with the formation of oxidized LDL (ox-LDL) with pro-inflammatory activity [33]. Macrophages express scavenger receptors (SR-A1) uptaking and phagocyting ox-LDL, thus becoming foam cells [32]. Activated ECs (endothelial cells) express adhesion molecules such as E- and P-selectins, vascular cell adhesion molecule 1 (VCAM-1), and intercellular adhesion molecule 1 (ICAM-1), which promote endothelial adhesion and transmigration into the intima of various leukocytes (monocytes, T and B-cells, and neutrophils) [31]. Monocytes present several membrane structures capable of interacting with adhesion molecules such as P-selectin glycoprotein ligand-1 (PSGL-1) or monocyte-expressed very late antigen-4 (VLA-4) [32]. Monocytes are activated to macrophages secreting pro-inflammatory cytokines such as IL-1, IL-6, IL-8, and TNF-α, thus triggering the inflammatory process [31,32]. As shown in previous studies, there is a direct correlation between HLD diet, hyperlipidemia, and inflammation [24,26,33,34].

Our study indicated that treatment with methotrexate reduces serum levels of hs-PCR and pro-inflammatory cytokines (IL-1β, IL-6, TNF-α), and significantly decreases the lipid profile (TC, LDL-C, TG), at the same time increasing the HDL-C level. Methotrexate is a systemic anti-inflammatory agent generally used for the treatment of rheumatoid arthritis and psoriasis. Epidemiological data suggest that low dose methotrexate (LDM) diminishes cardiovascular risk by lowering the serum levels of some pro-inflammatory cytokines (IL-1β, IL-6, TNF-α), C-reactive protein, and producing favorable changes in the lipid profile [35,36,37,38,39,40]. These results are consistent with other research papers published [35].

In the present study, infliximab administration leads to significant lipid profile changes, increasing the TC, LDL-C, TG, and HDL-C. Also, treatment with infliximab leads to a lower level of inflammatory markers, reducing TNFα, IL-1β, and IL-6 levels. TNF-α is a cytokine with a central role in inflammation, innate and adaptive immunity, apoptosis, and lipid metabolism [41]. TNF-α plays an essential role in developing atherosclerosis by promoting adhesion molecules’ expression on endothelial cells, inducing some critical inflammatory mediators and initiating the inflammatory cascade [42,43]. There is a direct link between TNF-α and lipid metabolism, interfering with TG and cholesterol metabolic pathways [44]. The first mechanism by which TNF-α increases TG’s plasma concentration is the inhibition of LPL activity, leading to a decrease clearance of TG-rich lipoproteins like very-low-density lipoprotein (VLDL), and thereby causing hypertriglyceridemia. The second known mechanism through which TNF-α interferes with TG’s metabolic pathway is the hepatic stimulation of TG synthesis by activating acetyl-CoA carboxylase, the key regulatory enzyme in fatty acid synthesis [44]. TNF-α also increases the plasma concentration of total cholesterol and the hepatic cholesterol synthesis, by stimulating β-hydroxy-β-methylglutaryl-CoA reductase activity (HMG-CoA), an essential regulatory enzyme regarding the cholesterol metabolic pathway [45].

So far, data regarding lipid profile alterations during treatment with infliximab are contradictory. Several studies reported that infliximab treatment is associated with an unfavorable lipid profile [46,47,48]. In contrast, others suggest that infliximab could have a reduced influence on the atherogenic lipid profile [49]. 

However, when comparing MTX to IFX, regarding lipid profile modifications, it seems that MTX provides significant antiatherogenic profile modifications highlighted by the decrease in total cholesterol, LDL-C, triglycerides, and overall atherosclerotic index (AI), while IFX presented with a pro-atherogenic profile. Although their anti-inflammatory properties are similar and protective in the present dyslipidemia model, the pro-atherogenic profile exhibited by IFX makes it a much less useful drug intervention.

It is well established that infliximab reduces the inflammatory process by inhibiting the TNFα pathway, leading to decreased pro-inflammatory cytokines such as IL-1β and IL-6, which are correlated with lower levels of hs-PCR, known as an independent predictor of cardiovascular risk [50]. Our findings were consistent with previous studies reported [46,47,48].

The HLD led to a high level of liver enzymes ALT and AST, indicating hepatocellular damage [51]. The administration of methotrexate produced no significant changes in the serum level of liver enzymes. Simultaneously, treatment with infliximab significantly increased AST and ALT plasma levels, suggesting that infliximab accentuated the hepato-cellular damage caused by the HLD.

Our findings revealed that GSH and GSH/GSSG ratio levels were significantly decreased in the HLD group compared to that in the SD group, while MDA and GSSG levels increased. These results suggest HLD decreased antioxidant capacity, leading to increased free radicals, and inducing lipid peroxidation and MDA overproduction [51]. Hyperlipidemia is correlated with the cell membrane’s altered physical properties, promoting an increase in the passage of free radicals from the mitochondrial electron transport chain or nicotinamide adenine dinucleotide phosphate (NADPH) activation oxidase [52]. Membrane lipids and lipoproteins are easily affected by oxidative stress since they are rich in polyunsaturated fatty acids. In the process of lipid peroxidation, a hydroperoxy group is internalized within the hydrophobic tails of unsaturated fatty acids. These alterations can lead to structural changes by disrupting the hydrophobic lipid–lipid, lipid–protein interactions or can generate hydroperoxyl radicals and reactive aldehyde derivates, which may produce secondary changes in the cell. The end products of lipid peroxidation, like malondialdehyde or 4-hydroxynonenal, can lead to protein damage by reactions with lysine amino groups, histidine imidazole groups, and cysteine sulphydryl group [53]. The increased levels of MDA could be attributed to increased ROS production or deficiency of antioxidant defense system. Superoxide dismutase (SOD) and GSHPx are the first lines of cellular defense against oxidative injury involved in the disposal of superoxide anions and hydrogen peroxide [54]. Glutathione is involved in many metabolic processes, presenting various cellular roles like effectively scavenging free radicals and other reactive oxygen species, eliminating lipid peroxides and hydrogen peroxides, and preventing multiple oxidation biomolecules [55]. Thus, inadequate detoxification of these reactive oxygen species by antioxidant enzymes can lead to an imbalance between antioxidant and oxidant systems. Excess production of free radicals depletes body antioxidants, leading to increased oxidative stress as seen in hypercholesterolemia conditions [56]. These findings are consistent with previous reports and suggest that HLD promotes intracellular oxidative stress [55]. 

Our results showed that the treatment with methotrexate and infliximab increased GSH and GSH/GSSG ratio levels and decreased MDA and GSSG levels, suggesting that these drugs can reduce oxidative stress and increase the antioxidant capacity. MDA and AA (acetaldehyde) are products of lipid peroxidation and merge with proteins and lipoproteins, creating a stable adduct malondialdehyde-acetaldehyde (MAA) [54]. Research has shown that MTX reduces ROS levels by preventing the formation of MAA adducts. MTX demonstrates natural antioxidants properties, capable of scavenging free radicals, especially superoxide (O^2−^), thus diminishing intracellular oxidative stress [55]. Some studies have shown that TNF-α may induce ROS generation by activating phagocytic NADPH oxidase in mitochondria and can also produce ROS from neutrophils, at the same time reducing glutathione and NADPH levels, two critical antioxidant factors. The molecular mechanism is still not entirely understood, so future studies are necessary to study the means through which infliximab produces changes in antioxidant levels [56,57]. Our findings are supported by previous studies reported in the literature, showing that the treatment with methotrexate and infliximab significantly reduces oxidant molecules and increases antioxidant capacity [57,58,59].

Furthermore, after HLD, the liver also showed significant accumulation of foam cells, non-alcoholic fatty liver (NAFLD) activity, and the evolution of steatohepatitis grading from mild to moderate, all these factors being direct risk factors in the onset of atherosclerosis [60,61].

Infliximab administration led to an increased collection of lipid droplets in the liver [62,63]. After treatment with infliximab, the histopathological analysis revealed that the percentage of foam cells in the liver increased and the NAS score remained high, together with high levels of liver enzymes (ALT and AST). This may be due to altered lipoprotein metabolism under treatment with infliximab [62,63]. Treatment with methotrexate slightly reduced total liver lipid accumulation, the NAS score, and the steatohepatitis grading without achieving statistical significance (*p* > 0.05). Although both drugs present beneficial effects concerning oxidative stress decrease, the differences regarding NAFLD risk recommend Mtx as the superior drug in terms of effectiveness and safety.

The aortic histopathological analysis revealed that the HLD, methotrexate, and infliximab did not produce differences regarding the aortic intima-media thickening in wild type rats.

In general, there were no significant differences regarding lipid profile, inflammation markers, liver function, and oxidative stress between the wild type and transgenic rats. However, transgenic rats displayed a much higher aortic sensitivity to HLD, with significantly higher aortic thickness (esp. media and intima) in this group compared to the wild type group, when exposed to HLD.

This increased sensitivity to HLD may be owed to the overexpression of the human beta-3 adrenoreceptor in the endothelium [22]. The aortic histopathological analysis of transgenic rats revealed that the group fed with HLD presented pathological changes such as aortic intima-media thickening, deposition of foam cells, a disordered arrangement of the nucleus, and the presence of smooth muscle cells and elastic fibers, all these being considered pathological features leading to atherosclerosis [60]. Overproduction of beta-3 receptors at the endothelial level presented an important factor promoting endothelial dysfunction and the production of specific lesions at this level in the context of HLD.

As shown before, these rats displayed a reduced eNOS production, associated with ^•^NO and O^2−^ overproduction, leading to endothelial dysfunction with impaired relaxation, increased aortic pressure, and ultimately leading to diastolic dysfunction [22].

Aortic histopathological section in transgenic rats showed that treatment with both methotrexate and infliximab improves the aortic wall structure, reducing the aortic intima-media thickness.

Regarding the analogy between Mtx, a conventional synthetic disease-modifying antirheumatic drug (DMARD), and Ifx, a biologic DMARD, we found that both drugs successfully decreased inflammation and oxidative stress markers in both types of animals, suggesting that these drugs could prevent the early onset of atherosclerosis. However, Mtx also normalized the lipid profile while Ifx induced extra-high TC, LDL-C, and TG levels. This difference is relatively explained by the mechanism of Ifx, particularly the blockade of TNF-α [44,45]. Furthermore, the histopathological analysis revealed that after treatment with methotrexate the steatohepatitis grading and the aorta intima-media thickness decreased. At the same time, infliximab produced a positive effect on aorta intima-media thickness without affecting the steatohepatitis grading.

## 5. Conclusions

In conclusion, our results showed that the HLD induced significant changes in plasma lipid levels, inflammation, and oxidative stress markers, thus initiating the main pathological processes that may lead to atherosclerosis. Methotrexate and infliximab reduced the inflammatory process and enhanced the antioxidant status in rats fed with HLD. However, infliximab increased the lipid profile and liver enzymes, while methotrexate effectively reduced the lipid profile but increased the liver enzymes, both drugs accentuating the hepatocellular damage caused by the HLD.

Overproduction of beta-3 receptors on the endothelial cells increased vascular reactivity to hyperlipidemia, inflammation, and oxidative stress. In this context, the vascular effects of methotrexate and infliximab could be observed.

Methotrexate and infliximab could represent potential therapeutic targets for treating dyslipidemia-induced vascular changes, leading to atherosclerosis by reducing the inflammatory process and oxidative stress. Still, the benefit–risk balance and the safety profile of these drugs must be carefully evaluated.

## Data Availability

Data is contained within the article.
